# The Novel Compound Sul-121 Preserves Endothelial Function and Inhibits Progression of Kidney Damage in Type 2 Diabetes Mellitus in Mice

**DOI:** 10.1038/s41598-017-11582-6

**Published:** 2017-09-11

**Authors:** S. P. H. Lambooy, A. Bidadkosh, D. Nakladal, A. van Buiten, R. A. T. Girgis, A. C. van der Graaf, T. J. Wiedenmann, R. A. Koster, P. Vogelaar, H. Buikema, R. H. Henning, L. E. Deelman

**Affiliations:** 1 Department of Clinical Pharmacy and Pharmacology, University of Groningen, University Medical Center Groningen, Groningen, The Netherlands; 2 Department of Pharmacology & Toxicology, Comenius University, Bratislava, Slovakia; 3 Sulfateq BV, Groningen, The Netherlands; 4 Department of Physiology, University of Heidelberg, Heidelberg, Germany

## Abstract

Diabetic nephropathy is still a common complication of type 2 diabetes mellitus (T2DM) and improvement of endothelial dysfunction (ED) and inhibition of reactive oxygen species (ROS) are considered important targets for new therapies. Recently, we developed a new class of compounds (Sul compounds) which inhibit mitochondrial ROS production. Here, we tested the therapeutic effects of Sul-121 on ED and kidney damage in experimental T2DM. Diabetic db/db and lean mice were implanted with osmotic pumps delivering Sul-121 (2.2 mg/kg/day) or vehicle from age 10 to 18 weeks. Albuminuria, blood pressure, endothelial mediated relaxation, renal histology, plasma creatinine, and H_2_O_2_ levels were assessed. Sul-121 prevented progression of albuminuria and attenuated kidney damage in db/db, as evidenced by lower glomerular fibronectin expression (~50%), decreased focal glomerular sclerosis score (~40%) and normalization of glomerular size and kidney weight. Further, Sul-121 restored endothelium mediated vasorelaxation through increased production of Nitric Oxide production and normalized plasma H_2_O_2_ levels. Sul-121 treatment in lean mice demonstrated no observable major side-effects, indicating that Sul-121 is well tolerated. Our data show that Sul-121 inhibits progression of diabetic kidney damage via a mechanism that involves restoration of endothelial function and attenuation of oxidative stress.

## Introduction

Type 2 diabetes (T2DM) is a growing public health problem and is associated with both micro- and macrovascular complications. Diabetic nephropathy (DN) is a frequent microvascular complication of T2DM and is currently the most common cause of End Stage Renal Disease (ESRD)^1^.

Endothelial dysfunction (ED), the impaired release of relaxing factors by the vascular endothelium, is known to be a key factor in the development of microvascular complications and end organ damage in patients with T2DM^2^. In the kidney, ED may not only contribute to impaired renal hemodynamics, but also causes increased protein leakage over the filtration barrier^3, 4^. The resulting albuminuria is now recognized as an early step in the development of diabetic nephropathy^5^.

The vascular endothelium releases several vasoactive substances, including nitric oxide (NO), prostaglandins and Endothelium-Derived Hyperpolarizing Factors (EDHF). In contrast to NO and the prostaglandins, EDHF has not been characterized as a single factor but rather is believed a combination of factors depending on the vascular bed studied. Potassium^6^, epoxyeicosatrienoic acids (EETs)^7^, C-type natriuretic peptide (CNP)^8, 9^ hydrogen peroxide (H_2_O_2_)^10, 11^ and more recently hydrogen sulfide (H_2_S)^12^ have all been identified as EDHFs.

In diabetes, oxidative stress is known to interact with the vasoactive substances released by the endothelium. Firstly, superoxides can react with NO, limiting the bioavailability of this vasodilator. Secondly, the generated peroxynitrite can react with tyrosine residues in caveolar proteins, resulting in nitric oxide synthase (NOS) uncoupling and impaired NO release^13^. As antioxidants have shown little clinical value in diabetic patients^14^, new approaches aiming to reduce ROS production in diabetes could provide additional treatment strategies for halting the progression of kidney disease in type 2 diabetes.

Recently, we developed modified 6-chromanols which represent a new class of pharmacological compounds (named Sul compounds) that preserve cell viability under conditions of hypothermia^15^. Interestingly, under these conditions, Sul-compounds preserve mitochondrial membrane potential through activation of mitochondrial membrane complexes I and IV. As a result, Sul compounds alleviate mitochondrial ROS production and preserve ATP production. In addition to the effects on mitochondria, Sul compounds have an antioxidant capacity due to the 6-chromanol group also found in alpha-tocopherol (Vitamin E) and Trolox. Although we reported these actions for the compound Sul-109^15^, identical results were obtained for Sul-121 (unpublished data). However, because of the better solubility of Sul-121 in the medium for the osmotic mini-pumps, Sul-121 was chosen over Sul-109 in the present study.

Given the effects of Sul compounds on mitochondrial ROS production, we hypothesized that Sul compounds may reduce ROS production and consequently improve ED in experimental diabetes. Here, we describe the effects of long term subcutaneous administration (8 weeks) of compound Sul-121 on the development of renal damage and endothelial dysfunction in the db/db model of type 2 diabetes.

## Results

### Animal characteristics

**Figure 1:**
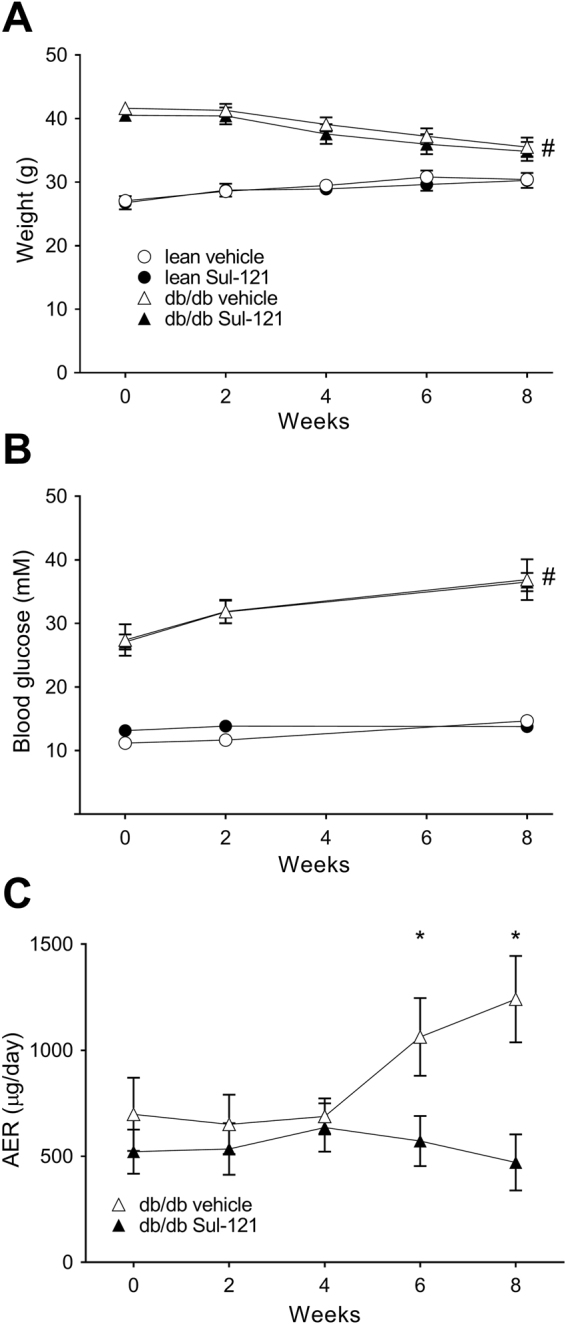
Effects of Sul-121 treatment on metabolic parameters and albuminuria in db/db mice and in lean mice. (**A**) Body weight and (**B**) non-fasting plasma glucose levels throughout the duration of the experiment. (**C**) The progression in urinary albumin excretion rate was attenuated by Sul-121 treatment in db/db Sul-121 vs db/db vehicle, from week 6 onwards. *p < 0.05 db/db Sul-121 vs db/db vehicle, ^#^p < 0.05 lean vs db/db.

Animal characteristics are shown in Fig. 1 and Table 1. As expected, db/db mice had a higher body weight compared to lean mice (Fig. 1A). db/db mice gradually lost weight as of week 4. However, Sul-121 did not affect body weight at any time point. Non-fasting blood glucose levels were significantly increased in the db/db mice compared to lean mice (Fig. 1B). During the course of the experiment, non-fasting blood glucose levels in the db/db animals rose from 27.5 ± 2.5 and 27.2 ± 1.3 g (db/db vehicle and db/db Sul-121, respectively) to 35.8 ± 2.4 and 35.3 ± 1.1 mM, indicating a severe and progressive diabetic state. Treatment with Sul-121 did not affect blood glucose levels.

**Table 1:** Animal characteristics.

Parameter	Lean vehicle (n = 8)	lean Sul-121 (n = 8)	db/db vehicle (n = 7)	db/db Sul-121 (n = 7)
body weight (g)	30.3 ± 0.6	30.2 ± 1.2	35.5 ± 1.5	34.8 ± 1.5
L kidney (mg)	227.2 ± 4.5	230.8 ± 7.8	261.7 ± 11.5^*^	233.1 ± 4.9#
R kidney (mg)	252.1 ± 6.0	264.1 ± 8.8	289.6 ± 7.8^*^	252.7 ± 7.3#
Liver (mg)	1356 ± 42	1388 ± 55	2194 ± 92^*^	2030 ± 63^*^
MABP (mmHg)(week 6)	74.1 ± 5.2	76.9 ± 6.5	85.7 ± 3.4*	87.5 ± 2.8*
24 h water consumption (weeks 2–8)	0.7 ± 5.2	0.8 ± 0.1	18.3 ± 1.2^*^	15.9 ± 1.5^*^
24 h urine production(weeks 2–8)	4.1 ± 0.2	3.7 ± 0.4	18.6 ± 1.2^*^	16.9 ± 1.6^*^
AER (μg/day)	13.5 ± 1.9	12.5 ± 1.7	1240.8 ± 203.9*	471.2 ± 132.2*#
plasma creat (μmol/l)	1.75 ± 0.17	1.24 ± 0.12*	1.96 ± 0.11*	2.16 ± 0.30*
plasma Sul-121 (μg/L)(week 2, median (IQR))	Non-detectable	50 (30–54)	Non-detectable	82 (66–235)†

Animal characteristics at week 8 of treatment unless stated otherwise. *p < 0.05 versus lean control, ^#^p < 0.05 versus db/db control, ^†^p < 0.05 vs lean Sul-121.

At termination, kidney and liver weights were measured (Table 1). In db/db vehicle, both left and right kidney weights were significantly increased (261.7 ± 11.5 and 289.6 ± 7.8 mg, respectively) compared to lean vehicle (227.2 ± 4.5 and 252.1 ± 6.0 mg, respectively). Sul-121 treatment normalized left and right kidney weights (233.1 ± 4.9 and 252.7 ± 7.3 mg, respectively). Liver weights were similarly increased in both db/db vehicle (2194 ± 92 mg) and db/db Sul-121 (2030 ± 63 mg) compared to lean vehicle (1356 ± 42 mg). Mean Arterial blood pressure was measured at week 6. Blood pressure was higher in db/db mice (85.7 ± 3.4 mmHg) compared to lean mice (74.1 ± 5.2 mmHg) and was not affected by Sul-121 treatment.

Plasma creatinine levels (Table 1) were similarly increased in both db/db vehicle (1.96 ± 0.11 μmol/l) and db/db Sul-121 (2.16 ± 0.30 μmol/l) compared to lean vehicle (1.75 ± 0.17 μmol/l). In lean Sul-121 mice, plasma creatinine levels were lower (1.24 ± 0.12 μmol/l) compared to lean vehicle.

### Sul-121 plasma levels in db/db and lean mice

Plasma Sul-121 levels were not normally distributed and ranged from 12 to 111 µg/l (median 50 µg/l) in lean Sul-121 mice (Table 1). Sul-121 levels were significantly higher in db/db Sul-121 than in lean Sul-121 (p < 0.05, range 55–274 µg/l, median 82 µg/l). Sul-121 levels in lean vehicle and db/db vehicle mice were all below detection threshold (<10 ug/l).

### The effects of Sul-121 on albuminuria

Urinary albumin excretion (AER) was measured throughout the duration of the experiment. Here, db/db vehicle mice showed an increase in AER from week 4 onwards reaching 1241 ± 204 µg/day by week 8. In db/db Sul-121, progression of AER was completely attenuated and AER remained stable at approximately 500 µg/day (Fig. 1C). AER in lean mice was measured at week 8 and amounted to 13.5 ± 1.9 and 12.5 ± 1.7 µg/day for lean vehicle and lean Sul-121 mice, respectively (Table 1).

### Glomerular damage and renal histology

**Figure 2:**
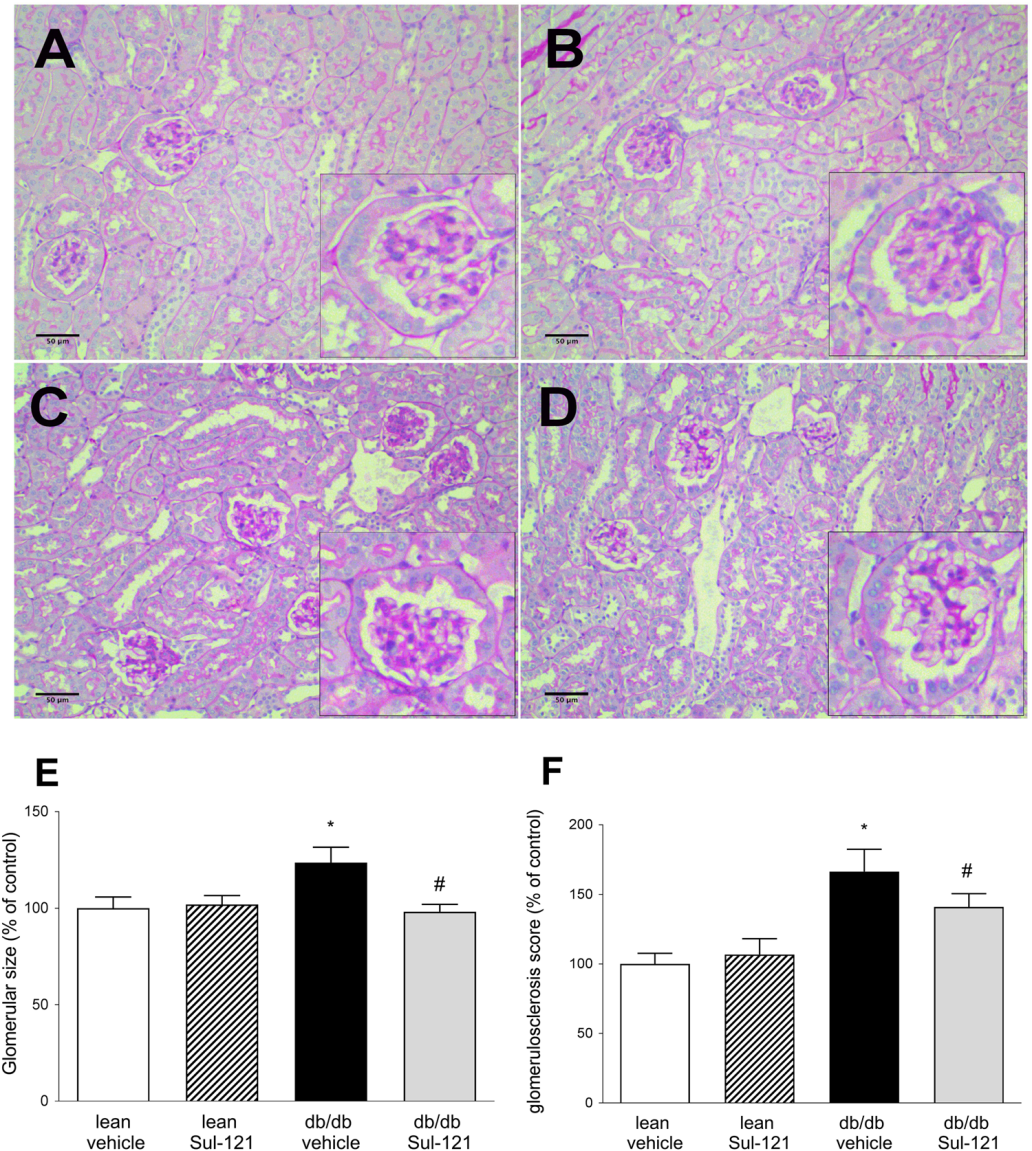
Effects of Sul 121 treatment on glomerular histology. Representative pictures of PAS stainings are shown for lean vehicle (**A**), lean Sul-121 (**B**), db/db vehicle (**C**) and db/db Sul-121 (**D**). Inserts show details of glomerular staining. (**E**) Glomerular size was increased in db/db vehicle and normalized in db/db Sul-121. (**F**) Glomerulosclerosis scores were increased in db/db vehicle and significantly reduced in db/db Sul-121. *p < 0.05 db/db vehicle vs lean vehicle, ^#^p < 0.05 db/db Sul-121 vs db/db vehicle.

As Sul-121 had a profound effect on albuminuria, we assessed damage to the glomerulus using histological staining and Western blotting. Periodic acid–Schiff stainings of kidney sections are shown in Fig. 2, panels *A-D*. Subsequently, glomerular size (Fig. 2E) and glomerulosclerosis scores (Fig. 2F) were determined. Glomerular size was increased in db/db vehicle (124 ± 8% of control), which was normalized in db/db Sul-121 (98 ± 4% of control). Similarly, glomerulosclerosis score was increased in db/db vehicle (166 ± 16% of control), which was significantly attenuated in db/db Sul-121 (140 ± 10% of control).

**Figure 3:**
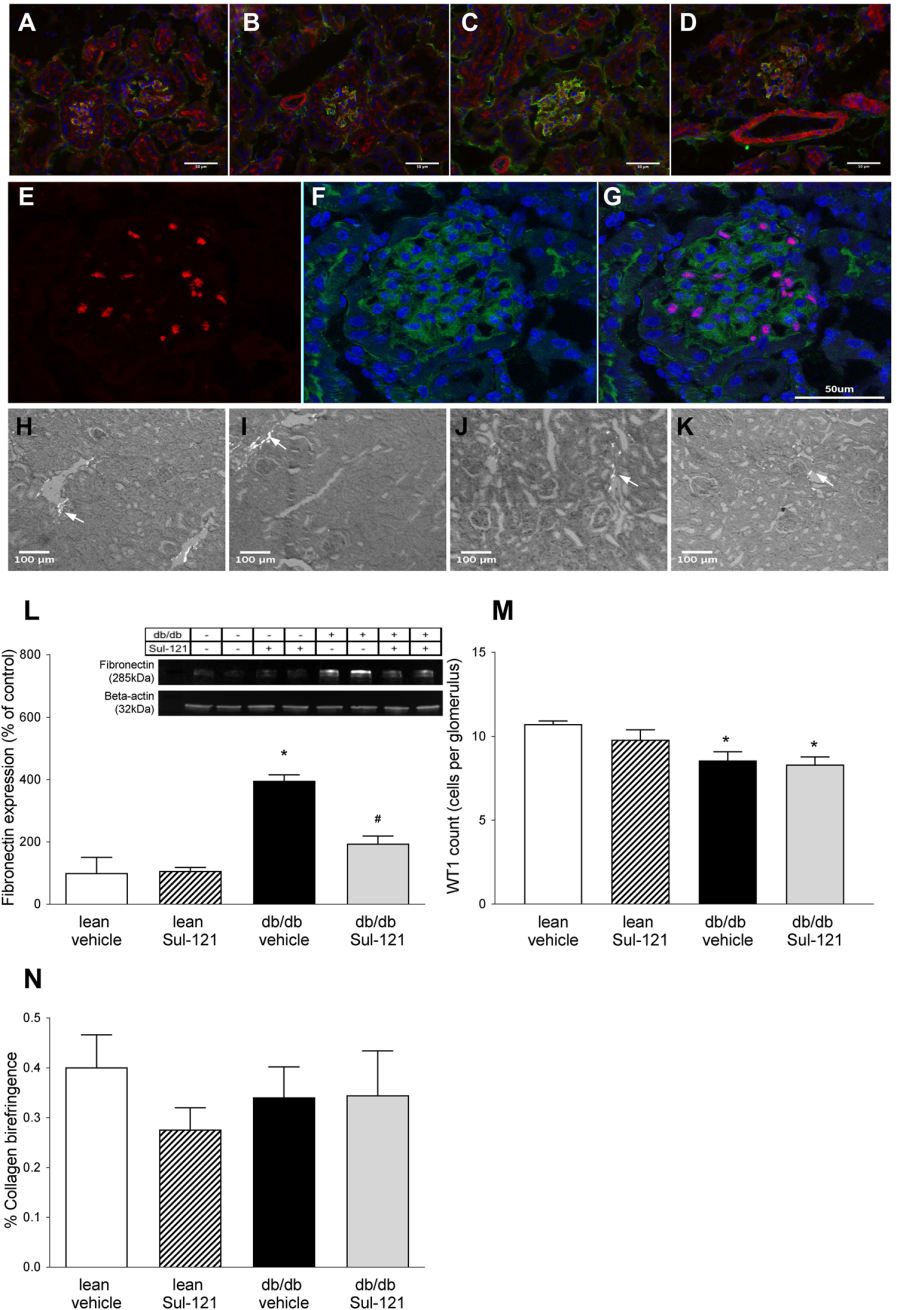
Effect of Sul-121 treatment on renal fibronectin, WT1 and Collagen I expression. (**A–D**) Stainings of fibronectin (green), actin (red), and dapi (blue) are shown for lean vehicle (**A**), lean Sul-121 (**B**), db/db vehicle (**C**) and db/db Sul-121 (**D**). Increased fibronection staining is observed only in db/db vehicle (**C**). (**E**,**F**) Stainings of podocyte marker WT1 (red), fibronectin (green) and dapi (blue) shown for lean vehicle only. (**E**) Nuclear staining of WT1. (**F**) Overlay of fibronectin and dapi. (**G**) Overlay of WT1, fibronectin and dapi. (**H**–**K**) Representative pictures of polarization contrast microscopy of collagen I (indicated by white arrows) in lean vehicle (**H**), lean Sul-121 (**I**), db/db vehicle(**J**) and db/db Sul-121(**K**). (**L**) Renal fibronectin expression was additionally examined using Western blotting. Insert showing representative cropped blots for fibronectin and beta-actin as loading control. Renal fibronectin was significantly upregulated in db/db vehicle. Sul-121 reduced fibronectin expression in db/db Sul-121. (**M**) Quantification of the average number of WT1 positive nuclei per glomerulus. Decreased numbers of WT1 positive cells were present in db/db vehicle and db/db Sul-121. (**N**) Quantification of polarization contrast microscopy of collagen I. *p < 0.05 db/db vehicle vs lean vehicle, ^#^p < 0.05 db/db Sul-121 vs db/db vehicle.

Glomerular damage was further assessed by immunohistochemistry. Expression of fibronectin, an extra cellular matrix (ECM) protein, was found predominantly in glomeruli (Fig. 3A–D) and was most pronounced in db/db vehicle (Fig. 3C). Fibronectin expression was further quantified by Western blotting in whole kidney homogenates and found to be significantly upregulated in db/db vehicle (396 ± 19% of control) compared to their lean vehicle (100 ± 51%) (Fig. 3L). In db/db Sul-121, the increased fibronectin expression was significantly attenuated (194 ± 24% of control).

Podycyte loss was assessed by immunostaining for podocyte markers WT1 and synaptopodin (Fig. 3E–G and Supplementary Fig. 1). WT1 staining was confined to the nucleus and podocyte numbers were determined by automated counting of the average number of WT1 positive cells per glomerulus (Fig. 3M). Compared to lean vehicle (10.7 ± 0.2 cells per glomerulus), the number of WT1 positive podocytes was significantly reduced in both db/db vehicle (8.6 ± 0.5 cells) and db/db Sul-121 (8.3 ± 0.5). For synaptopodin, a trend towards lower synaptopodin staining in db/db vehicle could be observed (Supplementary Fig. 1A,B). However, the decreased synaptopodin staining in db/db vehicle did not reach statistical significance (p = 0.13). Expression of slit diaphragm proteins was assessed by Western blotting for Nephrin (Supplementary Fig. 1C). No significant differences could be observed between groups.

**Figure 4:**
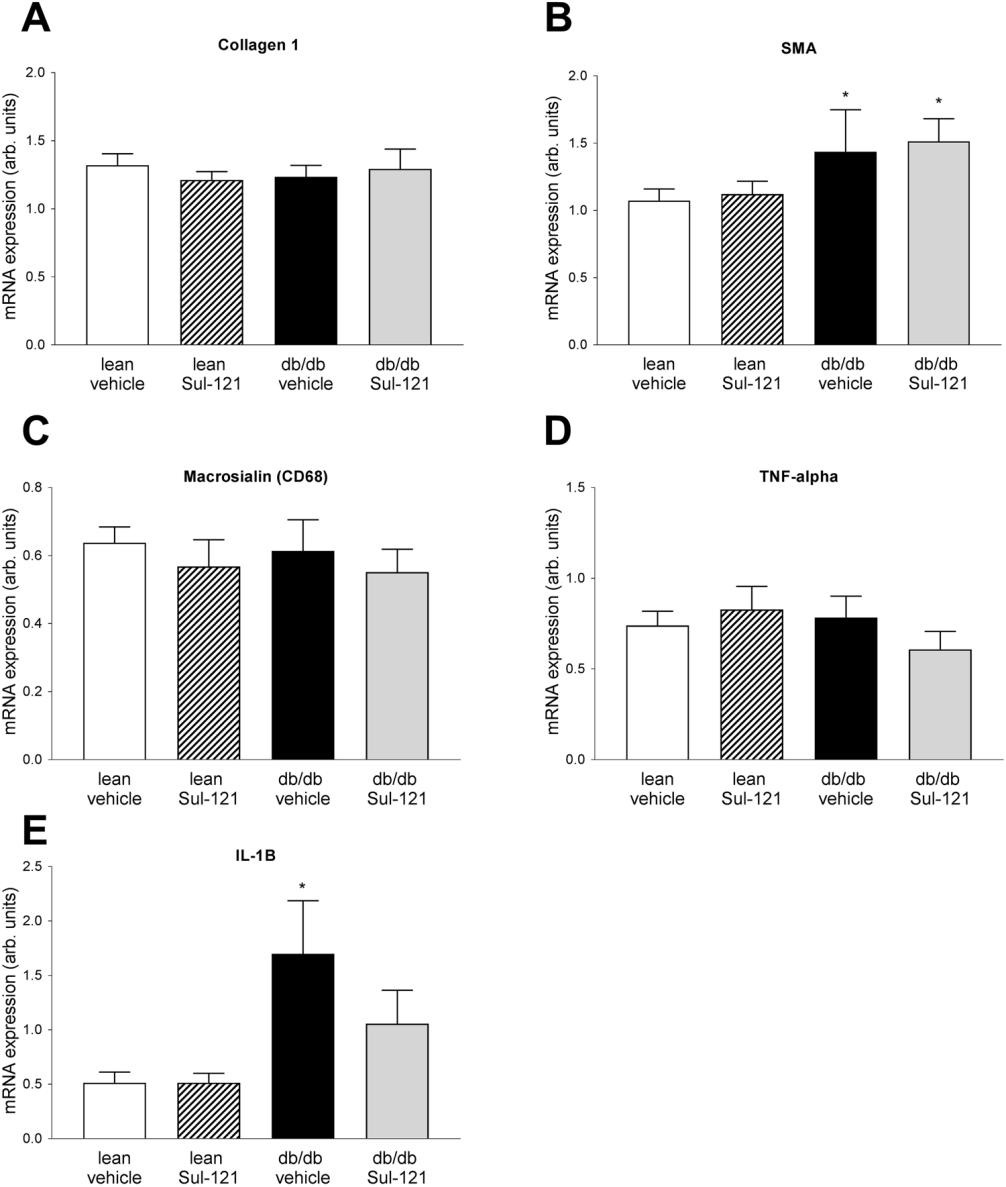
Effect of Sul-121 treatment on expression of markers of interstitial damage and inflammation. Expression of Collagen 1 (**A**), Smooth muscle actin (SMA) (**B**), Macrosialin (CD68) (**C**), TNF-alpha (**D**) and IL-1B (**E**) was assessed by real-time PCR. SMA expression was increased in db/db vehicle and db/db Sul-121(**B**). IL-1B was increased in db/db vehicle(**E**). *p < 0.05 db/db vehicle or db/db Sul-121 vs lean vehicle.

Sirius red staining and polarized microscopy was used to visualize collagen I as marker of renal interstitial damage (Fig. 3H–K). No differences in collagen I expression could be detected between groups (Fig. 3N). Additional markers for renal interstitial damage were assessed by real-time PCR (Fig. 4). Similar to the findings of the Sirius red staining, Collagen I mRNA expression was not different between groups (Fig. 4A). Smooth muscle actin expression (Fig. 4B) was increased in db/db vehicle (1.43 ± 0.31 arb. units) over lean vehicle (1.07 ± 0.09 arb. units), but not different in db/db Sul-121 (1.51 ± 0.16 arb. units). In addition, macrophage marker Macrosialin (CD68) and TNF-alpha expression was not different between groups (Fig. 4C,D). Interleukin Il-1B was upregulated in db/db vehicle (1.70 ± 0.48 arb. units) over lean vehicle (0.51 ± 0.10 arb. units) with a non-significant reduction in db/db Sul-121 (1.05 ± 0.31 arb. units) (Fig. 4E).

### Effects of Sul-121 treatment on reactive oxygen species

**Figure 5:**
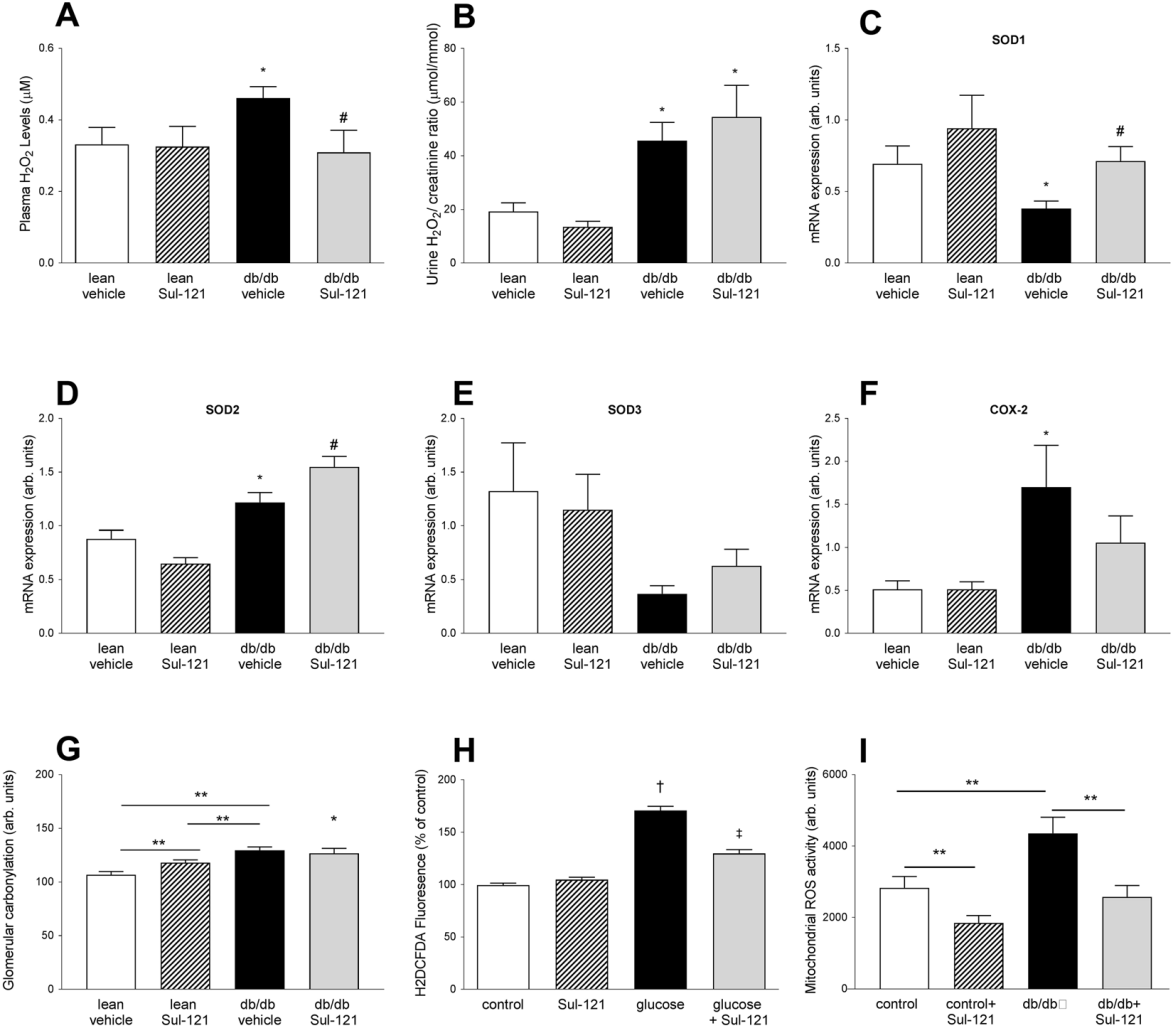
Effect of Sul-121 treatment on ROS status. (**A**) Plasma H_2_O_2_ levels are increased in db/db vehicle and normalized in db/db Sul-121) (**B**) Urinary H_2_O_2_ excretion is increased similarly in both db/db vehicle and db/db Sul-121. (**C**) SOD1 expression is decreased in db/db vehicle and normalized in db/db Sul-121. (**D**) SOD2 expression is increased in db/db vehicle and further increased in db/db Sul-121. (**E**) SOD3 expression was not significantly different between groups. (**F**) COX2 expression was increased in db/db vehicle. (**G**) Glomerular protein carbonylation was increased in lean-Sul-121 and further increased in db/db vehicle. Protein carbonylation in db/db Sul-121 was similarly increased as in db/db vehicle. (**H**) Effect of Sul-121 on *in-vitro* ROS production. Stimulation of cultured mesangial cells with high glucose/insulin increased ROS production which could be inhibited by Sul-121 pretreatment. (**I**) Acute effects of Sul-121 on renal mitochondrial ROS production *ex-vivo*. Increased mitochondrial ROS production in db/db was decreased by Sul-121. *p < 0.05 db/db vehicle vs lean vehicle, ^#^p < 0.05 db/db Sul-121 vs db/db, **p < 0.05 as indicated by horizontal line, ^†^p < 0.05 glucose vs control, ^‡^P < 0.05 glucose + Sul-121 vs glucose.

Hydrogen peroxide, a stable metabolite of ROS, was taken as a reference for ROS levels in plasma (Fig. 5A). Plasma H_2_O_2_ was significantly increased in db/db vehicle mice (0.46 ± 0.03 µM) compared to lean vehicle (0.33 ± 0.05 µM). Treatment of Sul-121 reduced H_2_O_2_ levels in db/db mice (0.33 ± 0.06 µM) to lean vehicle levels. Urinary H_2_O_2_ levels (Fig. 5B) were similarly increased in both db/db vehicle and db/db Sul-121 (45.6 ± 6.8 and 54.5 ± 11.8 μmol/mmol, respectively) over controls (19.2 ±3.2 μmol/mmol).

Furthermore, we examined the renal mRNA expression of superoxide dismutases SOD1, SOD2 and SOD3 (Fig. 5C–E). SOD1 expression was decreased in db/db vehicle (0.38 ± 0.05 arb. units) compared to lean vehicle (0.69 ± 0.12 arb. units) and was normalized in db/db Sul-121 (0.71 ± 0.09 arb. units). SOD2 expression was increased in db/db vehicle (1.22 ± 0.09 arb. units) compared to lean vehicle (0.88 ± 0.08 arb. units) and further increased in db/db Sul-121 (1.55 ± 0.10 arb. units). SOD3 expression demonstrated large variation with a trend towards increased SOD3 expression in db/db Sul121 (0.63 ± 0.17 arb. units) over db/db vehicle (0.37 ± 0.17 arb. units) although this did not reach statistical significance.

COX-2 expression (Fig. 5F) was increased in db/db vehicle (1.70 ± 0.49 arb. units) over lean vehicle (0.51 ± 0.10 arb. units). COX2 expression in db/db Sul-121 was intermediate (1.06 ± 0.33 arb. units) between lean vehicle and db/db vehicle and did not differ significantly from either group.

Glomerular protein carbonylation was determined in all groups (Fig. 5G and Supplementary Fig. 2). Surprisingly, lean Sul-121 showed increased protein carbonylation (118.0 ± 2.6 arb. units) over lean vehicle (106.8 ± 3.0 arb. units). In db/db vehicle, protein carbonylation was further increased (129.5 ± 3.2 arb. units), whereas this was not affected by Sul-121 treatment in db/db Sul-121 (126.0 ± 4.7 arb. units).

The effects of Sul-121 on ROS production were further studied in an *in-vitro* model of diabetes (Fig. 5H). For this, cultured mouse renal mesangial cells were exposed to conditions simulating type 2 diabetes (high glucose and insulin). Exposure to high glucose and insulin increased the fluorescence of the ROS probe H2DCFDA by approximately 70% (Fig. 5H). Interestingly, Sul-121 pre-treatment did not affect basal levels of intracellular ROS, but substantially inhibited the increase in intracellular ROS provoked by glucose and insulin (p < 0.05).

To assess the effects of Sul-121 on renal mitochondrial ROS production, mitochondria were isolated from kidneys from an additional group of lean and db/db mice. Mitochondria obtained from db/db mice showed increased ROS production (3956 ± 241 arb. units) compared to lean mice (2832 ± 315 arb. units)(Fig. 5I). Acute treatment with Sul-121 lowered mitochondrial ROS production to similar levels in both lean control (1849 ± 206 arb. units) and db/db (2302 ± 193 arb. units).

### Vascular function in diabetes and the effect of Sul-121

**Figure 6:**
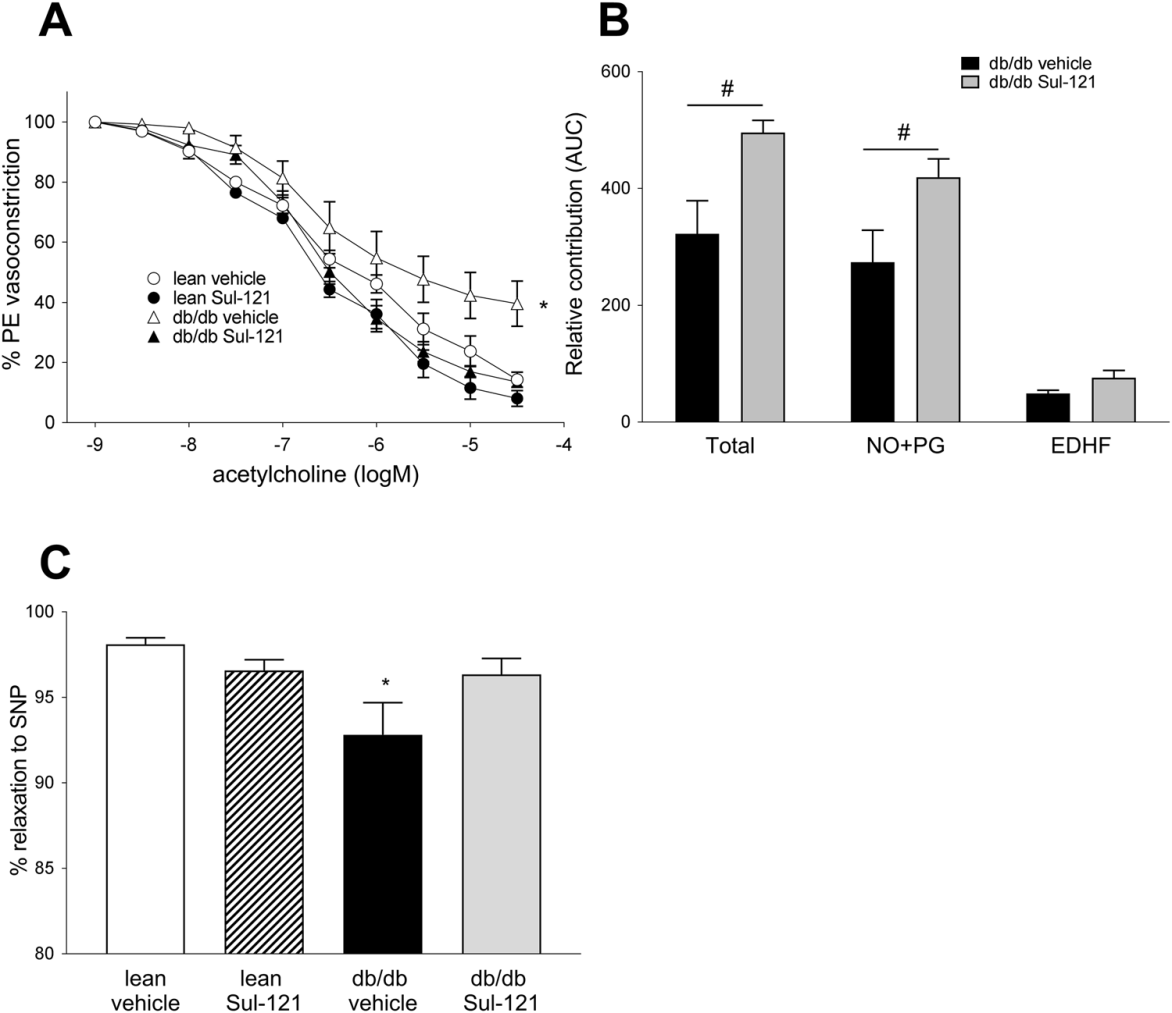
Sul-121 treatment restores endothelium mediated relaxation. (**A**) Relaxations to acetylcholine were reduced in aortic rings of db/db vehicle indicating endothelial dysfunction. Sul-121 restored relaxation to lean vehicle values. (**B**) Dissection of the components involved in relaxation demonstrated a significant increase of the combined NO + PG component in db/db Sul-121 vs db/db vehicle. *p < 0.05 db/db Sul-121 vs db/db vehicle. (**C**) Sensitivity of the aortic rings to the NO donor SNP demonstrated decreased NO sensitivity in db/db vehicle. *p < 0.05 db/db vehicle vs lean vehicle, ^#^p < 0.05 db/db Sul-121 vs db/db vehicle.

As ROS and diabetes have profound effects on endothelial function, we next established the effects of Sul-121 treatment on endothelial function of the mouse aorta. For this, mouse aortic rings were preconstricted with phenylephrine (PE) and subsequently relaxed with stepwise incremental concentrations of acetylcholine (ACh). Concentration effect curves were constructed (Fig. 6A), demonstrating an impaired relaxation to ACh in db/db vehicle compared to lean vehicle (max. relaxation 39.2 ± 6.5% and 9.2 ± 1.6% for db/db vehicle and lean vehicle, respectively). In db/db Sul-121 mice, vasorelaxation was completely restored to lean vehicle levels (11.3 ± 2.3%).

To further investigate the individual endothelial components involved in vascular relaxation, dose response curves were constructed using specific inhibitors for eNOS (L-NMMA) and cyclo-oxygenase (indomethacin). The relative contribution of each component was calculated (Fig. 6B). In db/db Sul-121 mice, total relaxation was improved mainly by an increase in the NO and prostaglandin components (274 ± 55 and 429 ± 31 AUC for db/db vehicle and db/db Sul-121, respectively). In addition, the EDHF component was higher in db/db Sul-121 (76 ± 12 AUC) compared to db/db vehicle (49 ± 6 AUC), although this did not reach statistical significance (p = 0.07).

Furthermore, we assessed the sensitivity of the smooth muscle cells to NO, using the NO donor sodium nitroprusside (SNP) (Fig. 6C). Relaxations of PE preconstricted aortic rings to SNP were significantly attenuated only in db/db vehicle (92.8 ± 1.9%) when compared to lean vehicle (98.1 ± 0.4%).

### Correlations

**Figure 7:**
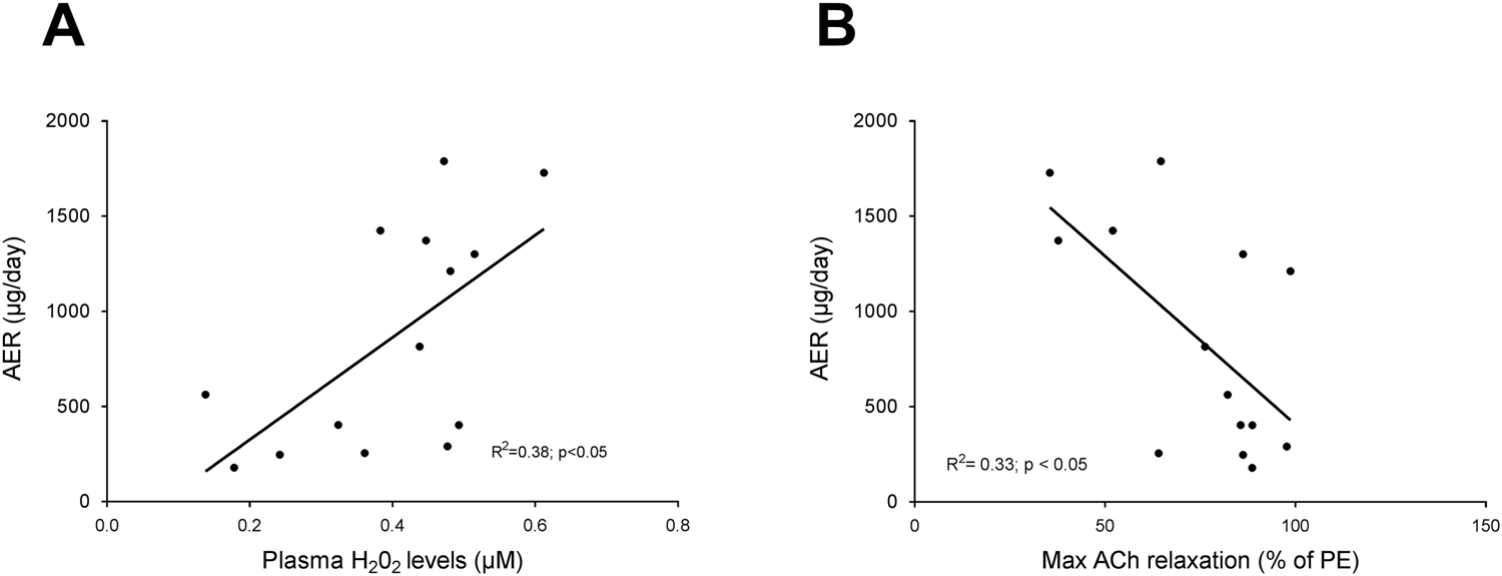
Albuminuria correlates with ROS status and endothelial function. (**A**) AER correlates significantly with plasma H_2_O_2_ concentrations (p < 0.05). (**B**) AER correlates significantly with maximal response to acetylcholine (p < 0.05).

To assess whether the improved ROS status or the normalization of endothelial function is contributing to the inhibition of renal damage progression in diabetic mice, we plotted correlations between albuminuria and plasma H_2_O_2_ levels (Fig. 7A) and between albuminuria and maximum endothelial mediated relaxation to ACh (Fig. 7B). Plasma H_2_O_2_ levels correlated positively with albuminuria (R^2^ = 0.38 p < 0.05) whereas maximal endothelial mediated relaxation correlated negatively with albuminuria (R^2^ = 0.33 p < 0.05) using data of the combined db/db vehicle and db/db Sul-121 groups.

## Discussion and Conclusion

The present study is the first to document the beneficial action of long-term subcutaneous infusion of the novel compound, Sul-121, in a model of experimental T2DM. We demonstrate that Sul-121 provides two major benefits. The first is the attenuated progression of albuminuria and the limited extent of diabetes related renal damage in the db/db model of T2DM. This was made evident by the slower advancement of albumin excretion rate, decreased focal glomerular sclerosis score, reduced glomerular ECM protein expression and normalization of glomerular size and kidney weight. Secondly, Sul-121 inhibited the development of endothelial dysfunction and normalized systemic ROS formation, which was supported by *in-vitro* experiments. Sul-121 treatment in lean mice had no major observable side-effects, indicating that it is a well-tolerated compound. Thus, Sul-121 inhibits the progression of experimental diabetic kidney damage via a mechanism that inhibits both oxidative stress and preserves endothelial function.

Importantly, treatment with Sul-121 did not affect metabolic and diabetic parameters. In both lean control and db/db mice, Sul-121 treatment had no effects on body weight, plasma glucose levels, water consumption or urine production, indicating that the protective effects of Sul-121 in diabetes were not mediated through a less severe diabetic state.

Rather, Sul-121 treatment had profound effects on the ROS status of the diabetic mice. First, Sul-121 normalized plasma H_2_O_2_ levels to levels of lean controls. H_2_O_2_ is considered to be the stable end product of short-lived reactive oxygen radicals and plasma H_2_O_2_ levels are determined by the kinetics of H_2_O_2_ production and elimination^16^. As urinary H_2_O_2_ excretion did not differ between db/db vehicle and db/db Sul-121, this indicates that Sul-121 indeed inhibits ROS production in diabetes. These findings were supported by our *in-vitro* experiments using the ROS probe H_2_DCFDA. There, Sul-121 was able to inhibit high glucose and insulin induced increase in ROS production in cultured mesangial cells, indicating that Sul-121 is able to decrease ROS production in response to high glucose. Further, we demonstrated that Sul-121 was able to inhibit increased ROS production by mitochondria obtained from kidneys of diabetic db/db mice. These findings are in line with a previous study performed in adipose tissue-derived stem cells^15^, confirming the inhibitory effects of Sul compounds on mitochondrial ROS production. In addition to inhibiting ROS production, Sul-121 stimulated renal expression of superoxide dismutases SOD1 and SOD2 through a yet unknown mechanism, which may have contributed to the decreased ROS status in db/db Sul-121. Finally, Sul-121 attenuated the up-regulation of renal COX2 expression in diabetic animals. COX2 expression is known to be induced by mitochondrial ROS production, and may contribute to disturbed autoregulation of glomerular blood flow in diabetes^17^. Taken together, these data demonstrate that Sul compounds can indeed reduce ROS production in diabetes confirming our hypothesis.

Further, Sul-121 had profound effects on endothelial mediated relaxation. In our study, vehicle treated db/db mice demonstrated substantial endothelial dysfunction, as evidenced by the impaired vasorelaxation to acetylcholine. Sul-121 treatment however, completely normalized endothelial function by restoring vasorelaxation of db/db mice to the level of lean control mice. Furthermore, Sul-121 treatment restored the NO sensitivity of the smooth muscle layer in diabetes. Endothelial dysfunction in the db/db models has been reported in numerous studies and is thought to be caused by reduced nitric oxide (NO) bioavailability as a consequence of oxidative stress. In keeping with this, the restored endothelial function in Sul-121 treated mice was associated with a normalization of plasma H_2_O_2_ levels in db/db mice. Furthermore, dissection of the endothelial factors involved in relaxation, demonstrated an increased contribution of NO and prostaglandins in the Sul-121 treated db/db mice. These data therefore indicate that normalization of oxidative stress and availability of NO underlie the normalization of endothelial function by Sul-121 in db/db mice.

Vascular endothelial dysfunction is often associated with renal endothelial dysfunction known to increase protein leakage over the filtration barrier, resulting in albuminuria^5^. Indeed, we found a significant correlation between vascular endothelial function and albuminuria in the present study. Furthermore, in addition to restoring vascular endothelial function, Sul-121 treatment halted the progression of albuminuria in db/db mice. Moreover, kidney hypertrophy, glomerular size, focal glomerular sclerosis scores and glomerular fibronectin expression were significantly lower in Sul-121 treated db/db over vehicle treated db/db mice, indicating a reduction in glomerular damage. Taken together these data indicate that the improved ROS status by Sul-121 treatment prevented vascular- and glomerular endothelial function, thereby attenuating the development of diabetic kidney damage.

In addition to damage to the endothelium, albuminuria may also be aggravated by loss of podocytes^18, 19^ and changes in the distribution and expression of slit-diaphragm proteins^20^. In the db/db model, loss of glucose sensitive podocytes is an early event and is already prominent at an age of 8 weeks, whereas the remaining majority of podocytes adapt to hyperglycemia and become resistant to diabetes-induced apoptosis^18^. Indeed, at the age of 10 weeks (at the start of treatment), the db/db mice in the present study already had increased albuminuria over lean mice, possibly reflecting this early loss of susceptible podocytes. Albuminuria in db/db mice progressed further at the age of 16 weeks (week 6 of treatment) which was prevented by Sul-121 treatment. However at the end of the study, podocyte numbers were found similarly reduced in both vehicle and Sul-121 treated db/db mice, indicating that the protective effects of Sul-121 treatment is not mediated through protection of the glucose resistant podocytes. Whether Sul-121 could protect the glucose sensitive podocytes at an earlier time point remains to be investigated in future studies.

Although the pharmacokinetics of Sul-121 are not fully known, the current study showed that Sul-121 plasma levels were significantly higher in db/db than in lean control mice. These differences cannot be simply explained by differences in body weight. As daily dosing was identical in lean control (low weight) and db/db mice (high weight), the opposite is expected, i.e. lower Sul-121 levels in db/db. Further, the administration of Sul-121 through osmotic mini pumps exclude that differences in oral absorption may underlie the higher Sul-121 levels in db/db. Moreover, db/db mice had a much higher urinary output, which seems to exclude renal clearance as the elimination route of Sul-121. Taken together, these data point towards a decreased hepatic clearance of Sul-121 in diabetes^21^ explaining its increased plasma concentration in db/db mice. Thus, it would be interesting in future studies to explore the pharmacodynamics/kinetics of Sul-121 to better understand its metabolism and clearance. Finally, we cannot exclude that the lower plasma levels of Sul-121 are contributing to the lack of major adverse effects in the lean mice.

Although the current study was carefully planned and executed, there are some limitations to consider. First, body weights in the db/db mice began to fall by week 4 of the experiment which corresponds with an age of fourteen weeks. This is considerably earlier than the decline in body weight at the age 5 to 6 months reported before^22^. Moreover, plasma glucose levels at the start of the experiment (age 10 weeks) were already at 28 mM which is considerably higher that the 15 mM reported for this age^23^. In addition, renal interstitial damage and inflammation were lower than expected from literature. At present, it is unclear why the db/db mice developed such a severe diabetic phenotype with mainly glomerular damage and low interstitial damage. Possibly, the relative high percentage of sugars in the institution’s standard RMH-B diet (4%) may be related to this. Regardless, this study demonstrates that Sul-121 treatment is effective even in a severely diabetic model.

Further, Sul-121 treatment caused a small (approximately 10%) but statistically significant increase in glomerular protein carbonylation in lean mice. This is surprising a no other adverse effects of Sul-121 was recorded in the present study. Furthermore, plasma H_2_O_2_ levels and expression of SOD1,2,3 and COX2 were all unaffected in Sul-121 treated lean mice, providing no indications for increased oxidative stress in Sul-121 treated lean mice. In addition, our *in-vitro* experiments did not show an effect of Sul-121 on ROS production in control cells. Alternatively, Sul-121 might interfere with protein decarbonylation. However, the molecular pathways of protein decarbonylation are still incompletely understood and whether Sul-121 interferes with electron donation to Glutaredoxin-1 or other enzymes involved in protein decarbonlyation^24^ needs to be determined in future studies. Nevertheless, the small increase in glomerular carbonylation in the absence of ROS activation appears to be without functional consequences for the kidney. In conclusion, Sul-121 limits the progression of experimental diabetic kidney damage via a mechanism that preserves endothelial function and reduces oxidative stress. Sul-121 may therefore represent a new therapeutic strategy to combat diseases characterized by endothelial dysfunction and oxidative stress, including diabetes, obesity and metabolic syndrome.

## Methods

### Animals

Experiments were approved by the Institutional Animal Care and Use Committee of the University of Groningen, Netherlands. Further, all methods were performed in accordance with the relevant guidelines and regulations. 16 male db/db (strain JAX000642) and 16 lean controls were purchased from Harlan, UK and were assigned into four groups: db/db vehicle (diabetic control), db/db Sul-121 (treated diabetic group), lean vehicle (non-diabetic control) and lean Sul-121 (to study effect of drug only). Osmotic mini pumps were implanted at the age of 10 weeks and replaced at 14 weeks. Mice were placed in metabolic cages for 24 hours every 2 weeks. Mice were housed conventionally with free access to standard pelleted food (RMH-B, Hope Farms, Woerden, Netherlands) and water. In the db/db vehicle group, one mouse became lethargic at week 1 and was prematurely terminated. In the db/db Sul-121 group, one mouse died during surgery at week 2 due to complications caused by the anaesthetic. Therefore, the number of animals in both db/db groups was reduced to n = 7.

An additional group of 5 db/db and 4 lean control mice were used for assessing renal mitochondrial ROS production.

### Chemicals and compounds

Vascular studies were performed using fresh Krebs bicarbonate solution. Cell culture grade DMSO was obtained from Sigma-Aldrich (Zwijndrecht, Netherlands). Sul-121 (6-hydroxy-2,5,7,8-tetramethylchroman-2-yl)(piperazin-1-yl)methanone) was supplied by Sulfateq bv, Groningen, Netherlands.

### Mini-pumps and dose of Sul-121

Previous unpublished experiments demonstrated that bolus infusions up to 30 mg/kg Sul-121 could be administered safely to mice. Therefore the maximal attainable dose of Sul-121 was chosen in the current study. For this, Alzet 2004 pumps were loaded with 200 µl of 12.6 g/l Sul-121 in 50% DMSO (or with 200 µl 50% DMSO solution as vehicle), resulting in a daily dose Sul-121 of 2.2 mg/kg/day for a 35gram mouse (range 1.8–2.82 mg/kg/day for the heaviest and lightest mouse in the study). Pumps were surgically implanted into a subcutaneous pocket in the dorsal midscapular area of the mouse.

### Sul-121 measurement

Blood samples for Sul-121 analysis were obtained two weeks after implantation of the first osmotic minipump. Sul-121 analysis was performed on an Agilent 6460 A (Santa Clara, Ca, USA) triple quadrupole LC-MS/MS system, with a combined Agilent 1200 series LC system. A HyPURITY® C18 analytical column from ThermoFisher Scientific (Waltham, MA, USA) was used for chromatographic separation by means of a gradient with a flow of 0.5 mL/min and a run time of 2.4 min. Sample preparation was performed by means of protein precipitation.

### Urinary measurements

Urinary glucose was measured by the glucose oxidase method with a specific electrode. Creatinine was measured by Jaffe method without deproteinization, DiaSys Diagnostic Systems, Holzheim, Germany. Albumin was determined manually using a mouse albumin Elisa kit, Abcam (ab108792), Cambridge, UK).

### Blood pressure measurement

Arterial blood pressure was measured in anaesthetized mice (2% isoflurane) by means of the tail-cuff method (PS-200A; Riken-Kaihatsu; Tokyo, Japan). For each animal, blood pressure values represented the mean of three to ten recordings.

### Myography

Thoracic aorta was surgically removed and stored in cold saline (4 °C). Aortas were cleaned from fat or connective tissue and cut into rings of 1.7–2.5 mm width. Rings were mounted in myograph baths (Danish Myo Technology, Aarhus, Denmark). Contractions were measured as cumulative concentration-response curves to phenylephrine followed by a single concentration of KCl (90 mM). Endothelium-dependent relaxation was assessed by obtaining concentration-response curves to ACh in rings precontracted with PE (1 μM) followed by stimulation with a high concentration of the NO donor sodium nitroprusside (SNP; 0.1 mM) to assess maximal endothelium-independent dilation. To examine the contribution of different EDRFs, inhibitors of PG (10 μM indomethacin) and NO synthesis (L-NMMA; 1 μM) were used. The remaining ACh-mediated relaxation was attributed to an unidentified EDHF.

### Histology and morphological analysis

For PAS and Sirius Red staining, Kidneys fixed in paraformaldehyde were cut into 4 μM sections, deparaffinised and stained with periodic acid-Schiff (PAS) or Sirius Red. Sirius Red staining was performed by incubating slides in 0.1% Sirius Red (manufacturer) for 60 minutes, washing twice with 1% acetic acid. Both PAS and Sirius Red Staining were subsequently dehydrated with 100% ethanol and cleared with xylene. Kidney sections stained with PAS were viewed under bright field illumination at 20x (Nikon Eclipse 50I). Glomerulosclerosis was scored in a blinded fashion by measuring mesangial expansion. For this, the circumference of the affected area in each glomerulus was drawn manually and the surface of this area was subsequently determined in Fiji version 2.00 (National Institutes of Health, USA). 10–50 glomeruli per kidney were analyzed. Kidney sections stained with Sirius Red were viewed under dark field illumination at 20x (TissueFAXs TissueGnostics). Whole scans were made and were analysed automatically according to method described by Street *et al*.^25^. For immunohistochemistry, kidney cryosections were cut into 4 micrometer sections, and stained for Nephrin (1:100, AF3159-SP, R&D research), WT1 (1:150, sc-192, Santa Cruz), Synaptopodin (1:100, sc-21537, Santa Cruz), anti-VWF (1:100, A008202, Dako), Fibronectin (1:100, sc-6952, Santa Cruz), dapi (nuclei) and Phalloidin (actin) (Texas Red-X, Thermofisher Scientific).

For the quantification of glomerular size and podocyte numbers, whole slide imaging was performed. Glomerular size was measured with ImageJ (NIH, v1.5i) only when the vascular pole was clearly visible. A minimum of 30 glomeruli per mouse was measured. The number of podocytes were quantified by the colocalization of DAPI and WT-1. A minimum of 32 glomeruli per mouse was counted.

For the localization of carbonylated proteins in kidney samples, the tissue had been fixed and was subsequently incubated with DNPH-solution diluted 1:100 in PBS. Afterwards unspecific antibody binding sides were blocked and the tissue stained with an anti-DNP-antibody (invitrogen, A11097, 1:100 dilution).

### Real-Time PCR

RNA from kidneys was isolated using a RNA isolation kit (NucleoSpin® RNA II, Macherey-Nagel GmbH & Co. KG). The integrity of RNA was determined using agarose gel electrophoresis, and the RNA concentration was measured spectrophotometrically at 260 nm. RNA (1 μg) was reversely transcribed into cDNA. Real-Time PCR was performed using Absolute^TM^ QPCR SYBR® Green ROX Mix (Westburg, Leusden, Netherlands), 400 nM of primer and 2,5 μl template DNA in a total volume of 25 μl. Quantitative real-time PCR was performed at 95 °C for 15 minutes followed by 40 cycles of denaturing at 95 °C for 15 seconds and annealing/ extending at 60 °C for 1 minute. Real-Time PCR for beta actin (forward primer: 5′-CGCCACCAGTTCGCCATGGA-3′, reverse primer: 5′-TACAGCCCGGGGAGCATCGT-3′), SOD1 (forward primer: 5′-AAGGCCGTGTGCGTGCTGAA-3′, reverse primer: 5′-ATCCGCTGGACCGCCATGTT-3′), SOD2 (forward primer: 5′-GGCGACCTACGTGAACAATCT-3′, reverse primer: 5′-TTGATAGCCTCCAGCAACTC-3′), SOD3 (forward primer: 5′-GACCGGCTTGACCTGGTTGA-3′, reverse primer: 5′-CCACGAAGTTGACGAAGTCC-3′), COX2 (forward primer: 5′-CTGTATCCCGCCCTGCTGG-3′, reverse primer: 5′-AAGACAGCCACCATCAACG-3′), CD68 (forward primer: 5′-AAGCAGCACAGTGGACATTC-3′, reverse primer: 5′-ATGATGAGAGGCAGCAAGAG-3′), Il-1B (forward primer: 5′-CTGTGGCAGCTACCTATGTC-3′, reverse primer: 5′-CACACTAGCAGGTCGTCATC-3′), TNF-a (forward primer: 5′-ACAAAGGTGCCGCTAACCACATGT-3′, reverse primer: 5′-ATGCTGCTGTTTCAGTCGAAGGCA-3′), SMA (forward primer: 5′-CCATCATGCGTCTGGACTTG-3′, reverse primer: 5′-AATCTCACGCTCGGCAGTA-3′) and Collagen-1 (forward primer: 5′-GGCCATTGGT GGTGCTGAC-3′, reverse primer: 5′-TAAAGGAGGAAACGGCAAAG-3′) was performed using Absolute^TM^ QPCR SYBR® Green ROX Mix (Westburg, Leusden, Netherlands), 400 nM of each primer and 2 μl template DNA in a total volume of 10 μl. Quantitative real-time PCR was performed at 95 °C for 15 minutes followed by 40 cycles of denaturing at 95 °C for 15 seconds and annealing/extending at 60 °C for 30 seconds, 75 °C for 30 seconds. PCR product specificity and purity were evaluated by generating a dissociation curve following the manufacturer’s recommendations.

### Western blotting

Frozen kidneys tissues were homogenized in cold RIPA with protease inhibitors and protein concentrations were determined by the Bio Rad DC protein assay. Proteins were separated by gel electrophoresis and transferred onto nitrocellulose membrane. Antibodies used for detection: beta-actin (sc-47778, Santa Cruz), Nephrin (AF3159-SP, R&D research), Fibronectin (sc-6952, Santa Cruz, Heidelberg, Germany), Protein expression was detected by a GeneGnome and images were analysed with Fiji.

### Plasma measurements

Plasma creatinine levels were determined using a Cayman Creatinine (serum) Colorimetric Assay Kit (Cayman, Ann Arbor, USA). Plasma hydrogen peroxide levels were determined using an Amplex Red H_2_O_2_ kit, Life technologies (A22188), Leusden, Netherlands.

### Cell culture and ROS production

Mouse mesangial cells (MMC) were cultured in DMEM containing penicillin and streptomycin supplemented with 10% fetal calf serum. ROS production was measured in 96 wells plates using the probe H2DCF-DA (C2938; Molecular probes). MMCs were first incubated in DMEM containing 1 g/l glucose 24 hours. Near confluent mesangial cells were loaded with dye for 1 hour at 37 °C. Afterwards, cells were washed and incubated with or without Sul-121 (100 nM) for 30 minutes. ROS production was stimulated by incubation in high glucose (4.5 g/l) and insulin (0.1 unit/ml) for 1 hour. Fluorescence was measured using a Synergy H4 microplate reader.

Renal mitochondrial ROS production was measured in an additional cohort of lean (n = 4) and db/db (n = 5) mice. For this, the kidney was excised and immediately stored in cold saline for no more than 2 hours. Subsequently, the kidney was cut into smaller pieces and a 100 mg kidney fragment was transferred into an eppendorf vial containing 0.5 ml of cold extraction buffer (10 mM HEPES pH 7.5, 200 mM mannitol, 70 mM sucrose and 1 mM EGTA). The tissue was homogenized and subsequently centrifuged at 600*g for 5 minutes. The supernatant was transferred to a new tube and centrifuged at 11.000 g for 10 minutes. The pellet was resuspended in 0.5 ml extraction buffer and the two centrifugation steps described above, were repeated. The resulting pellet was resuspended in storage buffer (10 mM HEPES pH 7.5, 250 mM sucrose, 1 mM ATP, 80 µM ADP, 5 mM sodium succinate, 2 mM K_2_HPO_4_ and 1 mM DTT). Protein concentrations were determined by a DC protein assay (Biorad). For measuring mitochondrial ROS production, 2 µg of mitochondria was transferred into 50 µl assay buffer (20 mM MOPS pH7.5, 110 mM KCl, 10 mM ATP, 10 mM KCl, 10 mM sodium pyruvate and 1 mM EGTA) supplemented with 100 µM Amplex Red en 0.2U/ml HRP (Invitrogen). To evaluate the acute effects of SUL-121 on mitochondrial ROS production, 1 µM of Sul-121 was added to the reaction mixture. The Amplex Red reaction was performed at 37 °C for two hours. Fluorescent detection was performed at 540/590 nm in a Biotek Synergy 4 plate reader.

### Data analysis and calculations

Data are presented as means ± SE, and n refers to the number of animals in each group. Statistical analysis was done with SPSS version 22 for Windows (SPSS, Chicago, IL). Differences between full concentration-response curves were tested with repetitive measures ANOVA; differences between points were tested with one-way ANOVA. P values of <0.05 (two tailed) were considered statistically significant.

## Electronic supplementary material

Dataset 1
