# Supplementary material for: The Novel Compound Sul-121 Preserves Endothelial Function and Inhibits Progression of Kidney Damage in Type 2 Diabetes Mellitus in Mice
Source: Sci Rep. 2017 Sep 11;7:11165. doi: 10.1038/s41598-017-11582-6 (PMC5593963; doi:10.1038/s41598-017-11582-6)

**The novel compound Sul-121 preserves endothelial function and inhibits progression of kidney damage in type 2 diabetes mellitus in mice.**

Authors: Lambooy SPH^1^, Bidadkosh A^1^, van Buiten A^1^, Girgis RAT^1^, van der Graaf AC^2^, Wiedenmann TJ^3^, Koster R^1^, Vogelaar P^2^, Buikema H^1^, Henning RH^1^, Deelman LE^1^*

Affiliation(s):

^1^Department of Clinical Pharmacy and Pharmacology, University of Groningen, University Medical Center Groningen, Groningen, the Netherlands.

^2^Sulfateq BV, Groningen, the Netherlands

^3^Department of Physiology, University of Heidelberg, Heidelberg, Germany


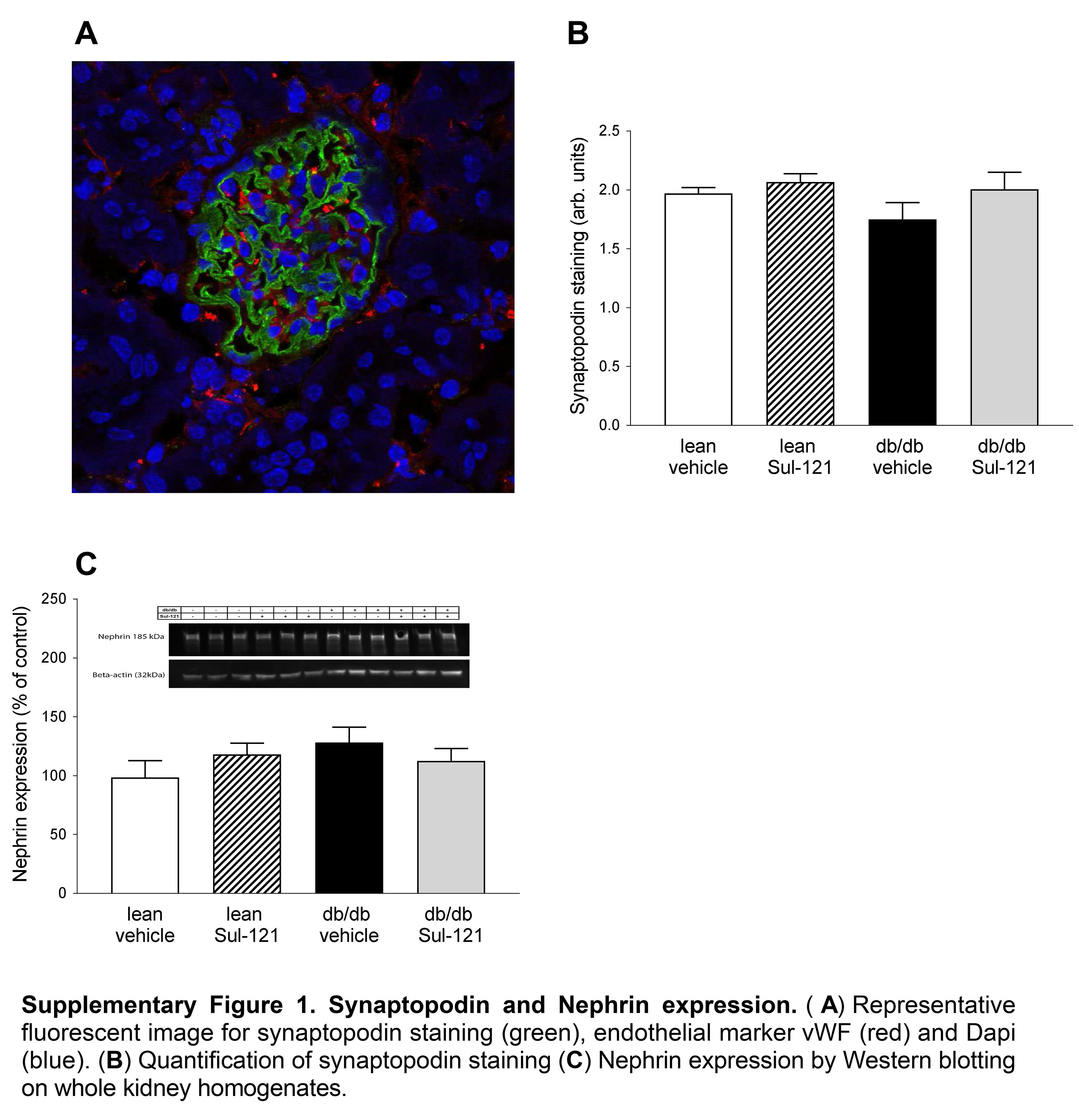


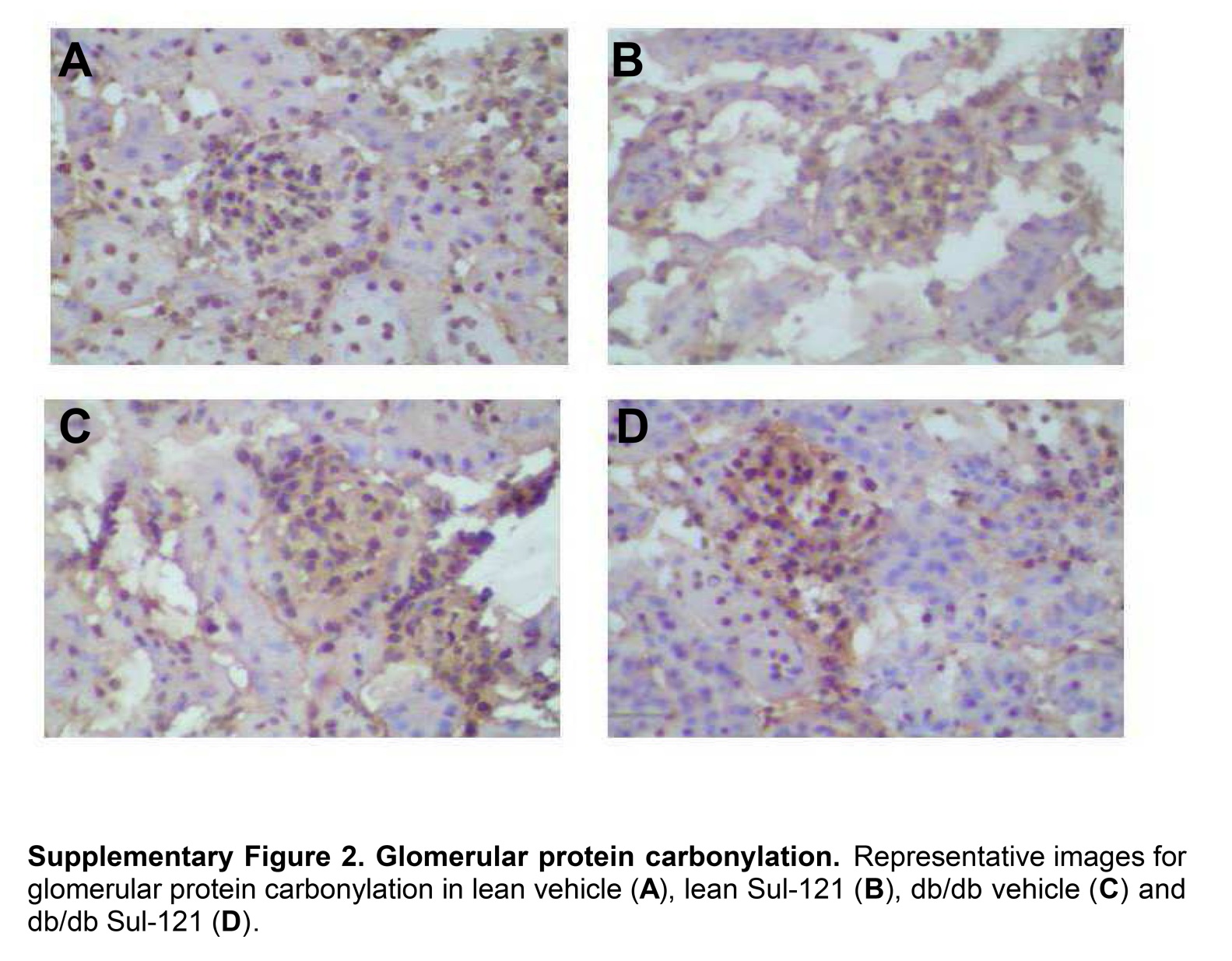

Supplement: Supplementary file 1 — Dataset 1 [file 41598_2017_11582_MOESM1_ESM.doc]
